# Common Variants of Homocysteine Metabolism Pathway Genes and Risk of Type 2 Diabetes and Related Traits in Indians

**DOI:** 10.1155/2012/960318

**Published:** 2011-09-25

**Authors:** Ganesh Chauhan, Ismeet Kaur, Rubina Tabassum, Om Prakash Dwivedi, Saurabh Ghosh, Nikhil Tandon, Dwaipayan Bharadwaj

**Affiliations:** ^1^Genomics and Molecular Medicine Unit, CSIR-Institute of Genomics and Integrative Biology, New Delhi 110 007, India; ^2^Human Genetics Unit, Indian Statistical Institute, Kolkata 700 108, India; ^3^Department of Endocrinology, All India Institute of Medical Sciences, New Delhi 110 029, India

## Abstract

Hyperhomocysteinemia, a risk factor for cardiovascular disorder, obesity, and type 2 diabetes, is prevalent among Indians who are at high risk of these metabolic disorders. We evaluated association of common variants of genes involved in homocysteine metabolism or its levels with type 2 diabetes, obesity, and related traits in North Indians. We genotyped 90 variants in initial phase (2.115 subjects) and replicated top signals in an independent sample set (2.085 subjects). The variant *MTHFR*-rs1801133 was the top signal for association with type 2 diabetes (OR = 0.78 (95%  CI = 0.67–0.92), *P* = 0.003) and was also associated with 2 h postload plasma glucose (*P* = 0.04), high-density lipoprotein cholesterol (*P* = 0.004), and total cholesterol (*P* = 0.01) in control subjects. These associations were neither replicated nor significant after meta-analysis. Studies involving a larger study population and different ethnic groups are required before ruling out the role of these important candidate genes in type 2 diabetes, obesity, and related traits.

## 1. Introduction

Homocysteine, a thiol containing amino acid, has emerged as a determinant for many complex metabolic disorders in the past few years. Hyperhomocysteinemia is a well-known independent risk factor for cardiovascular diseases [1, 2] and is also associated with diabetic complications [3–6], obesity, [7, 8] and metabolic syndrome [9]. Hyperhomocysteinemia may lead to any of the above metabolic disorders by elicitation of oxidative stress [10], systemic inflammation, [11] and/or endothelial dysfunction [12]. These factors are known to promote insulin resistance and *β*-cell dysfunction, two important underlying causes for type 2 diabetes [13]. Indians are at high risk for these metabolic disorders, as reflected by high prevalence of type 2 diabetes patients and metabolic syndrome [14, 15]. The observed trend of hyperhomocysteinemia among Indians [16] indicates the possibility of aberrant homocysteine metabolism with increased risk of metabolic disorders in Indian population. Hence, it becomes important to examine the association of variants of genes involved in homocysteine metabolism or modulating plasma homocysteine level with type 2 diabetes and related traits in Indians. 

Previous studies suggested contribution of variants in homocysteine metabolism pathway genes in susceptibility to obesity, type 2 diabetes, or related traits [17–19]. Methylene tetrahydrofolate reductase (*MTHFR*) is shown to act synergistically with angiotensin-I-converting enzyme (*ACE*) to modulate type 2 diabetes risk [17]. The C677T polymorphism (rs1801133) of *MTHFR* is the most studied genetic variation and is associated with hyperhomocysteinemia [20]. Many studies have also reported association of C677T with type 2 diabetes and related complications [21–26]. Variants of *MTHFR* and other homocysteine metabolism pathway genes like* MTR* and *MTRR* have also been shown to be associated with obesity [27]. However, little insight is available in this regard from limited reports showing association of variants in homocysteine metabolism pathway genes with plasma homocysteine levels in Indians [28, 29]. Here, we assessed association of 90 SNPs in 18 genes, from homocysteine metabolism pathway or those associated with homocysteine levels, with type 2 diabetes, obesity, and related traits in 4.200 North Indians.

## 2. Materials and Methods

### 2.1. Subjects

The study was performed in two phases: evaluation of 90 SNPs for association with type 2 diabetes and related traits in the first phase and replication of top signal in the second phase. All study participants were unrelated, urban dwellers of Indo-European ethnicity from North India. All nondiabetic controls were recruited through “diabetes awareness camps” conducted in Delhi and National Capital Region, while type 2 diabetes patients were recruited from Endocrinology Clinic, All India Institute of Medical Sciences, New Delhi. Study population for the first phase consisted of 2,115 subjects comprising of 1.073 type 2 diabetes patients (from clinics) and 1.042 nondiabetic controls. Replication phase comprised of an independent sample set of 2.085 subjects including 1.047 type 2 diabetes patients and 1.308 nondiabetic controls. Subjects were characterized as type 2 diabetes patients and controls based on inclusion and exclusion criteria described previously [30]. Briefly, type 2 diabetes was defined by the WHO criteria [31], while criteria for non-diabetic controls were as follows: age ≥40 years, HbA1c <6.0%, fasting plasma glucose <6.11 mmol/L, and no family history of diabetes.

Anthropometric and biochemical estimations were performed as described earlier [30]. The measured variables included height, weight, waist circumferences, hip circumferences, systolic and diastolic blood pressure, body mass index (BMI) and waist to hip ratio (WHR), glycosylated haemoglobin 1c (HbA1c), fasting plasma glucose (FPG), 2h postload plasma glucose (2h PPG), fasting plasma insulin (FPI), C-reactive protein (CRP), C-peptide, total cholesterol (TC), high-density lipoprotein cholesterol (HDL-C), low density lipoprotein cholesterol (LDL-C), triglyceride (TG), blood urea, and creatinine. Subjects with BMI < 23 kg/m^2^ were classified as normal weight (NW), whereas subjects with BMI ≥ 23 kg/m^2^ were considered as overweight/obese (OW/OB), according to WHO Expert Committee recommendations for Asians [32]. Homeostasis model assessments of insulin resistance (HOMA-IR), *β*-cell function (HOMA-B), and insulin sensitivity (HOMA-S) were obtained using formulae as reported previously [33, 34].

The study was approved by ethics committee of participating institutions and was conducted in accordance with the principles of Helsinki Declaration. Written informed consent was obtained from each study subject.

### 2.2. Genotyping

We selected 79 SNPs from 15 genes which are involved in homocysteine metabolism pathway and 11 SNPs from 2 genes (*NOS3* and *ACE*) reported to be associated with homocysteine levels [17, 35] (see Table  1 in Supplementary Material available online at: 10.1155/2012/960318). SNPs in functional regions, with minor allele frequency (MAF) >0.05, polymorphic in at least two populations and tag SNPs were preferred. Genotyping in the first phase was performed using Illumina Golden Gate assay. Stringent quality control (QC) criteria were applied as provided previously [36]. Also, 7.2% (147 samples) of total samples were genotyped in duplicate, and an error rate < 0.01 was figured. Of the 78 QC passed SNPs, those with MAF <0.05 (*N* = 9) or deviating from Hardy-Weinberg equilibrium (HWE) in control subjects (*N* = 2)  (*P* < 6.41 × 10^−4^, 0.05/78) were also excluded. Consequently, a total of 67 SNPs were considered for further association analyses.

In replication phase, we genotyped the top signal of first phase, rs1801133 (*MTHFR*), using iPLEX on a MassARRAY System (Sequenom, San Deigo, Calif, USA). The average genotyping success rate was 97% with >99.8% consistency rate in genotype calls in 5% duplicates.

### 2.3. Statistical Analysis

Genotype distributions were analyzed for deviation from HWE using *χ* ^2^ test. Association of SNPs with type 2 diabetes and obesity was determined by logistic regression assuming log additive model. Linear regression was performed under log additive model for association analysis of SNPs with quantitative traits in control subjects. The analyses were adjusted for sex, age, and BMI as appropriate. Odds ratios (ORs), *β*, and 95% confidence intervals (CI,) were calculated with respect to minor allele. To assess difference in allele frequency of the two study populations, equality of proportions *Z*-test was applied. Meta-analysis under fixed and random effect models was done by merging summary statistics of two phases. After correcting for multiple testing (Bonferroni correction), a *P *value <3.32 × 10^−5^  (*α* = 0.05/(58 × 26)) was considered significant (58 independent SNPs and 26 traits). Power of study was calculated assuming 10% disease risk at *α* = 0.05 under log additive model using Quanto (http://hydra.usc.edu/gxe/). Statistical analyses were performed using SPSS version 17.0 (SPSS, Chicago, Ill), Stata 10.1 (Stata Corporation, College Station, Tex, United States), and PLINK (http://pngu.mgh.harvard.edu/purcell/plink/) unless specified otherwise.

## 3. Results

Clinical characters of the study subjects (phase 1 and phase 2) are provided in Table 1. The study had >82.9% power to detect association of variants with allele frequency of 0.20 and genetic effect size of 1.25. Allele frequency of controls and cases of the initial and replication phase for SNP rs1801133 did not differ significantly. The allelic and genotypic distribution of SNPs for type 2 diabetes patients and non-diabetic control subjects recruited in the first phase are provided in Supplementary Table  1, whereas details for normal weight (NW) and overweight/obese (OW/OB) subjects are presented in Supplementary Table  2. 

Association analysis results of all the SNPs with type 2 diabetes are presented in Supplementary Table 3. *MTHFR* variant rs1801133 showed strongest association with type 2 diabetes in initial phase (OR=0.78 (95% CI 0.67–0.92), *P* = 0.003) (Table 2). However, this could not be replicated in the second phase (OR=1.01 (95% CI 0.87–1.19), *P* = 0.86). Meta-analysis also failed to detect any association of rs1801133 with type 2 diabetes (*P* random effect (*P*_*r*_) = 0.38, *P* fixed effect (*P*_*f*_) = 0.05) (Table 3).

Our previous studies have indicated influence of obesity status on association of genetic variants with type 2 diabetes in North Indians [30, 37]. Thus, we stratified our subjects into NW and OW/OB groups and assessed association with type 2 diabetes in these subgroups. Among NW subjects, we observed nominal association of *MTHFR*-rs1801133, *MTHFR*-rs9651118, *CHDH-*rs4563403, *CBS*-rs706208, and *MTHFD1L-*rs1555179 with type 2 diabetes (*P* value range= 0.002–0.04) (Table 2). On the other hand, in OW/OB individuals, *MTHFD1*-rs1076991 (OR = 0.81 (95% CI 0.69–0.95) *P* = 0.01) and *TCN2*-rs1801198 (OR = 0.85 (95% CI 0.72–0.99), *P* = 0.04) showed nominal association with type 2 diabetes. In the second phase, association of *MTHFR*-rs1801133 with type 2 diabetes in NW subjects could not be replicated either in the second phase (*P* = 0.53) or upon Meta-analysis (*P*_*r*_/*P*_*f*_ = 0.52/0.07) (Table 3).

Further, we investigated association of these SNPs with obesity status (OW/OB versus NW) in non-diabetic control subjects (Supplementary Table  4). Only a nominal association of *CHDH*-rs4563403 (OR = 0.69 (95% CI 0.52–0.92), *P* = 0.01), *TCN2-*rs1801198 (OR = 1.24  (95% CI 1.04–1.48), *P* = 0.02), and *MTR*-rs16834521 (OR = 0.82 (95% CI 0.68–0.99), *P* = 0.04) was observed in the first phase (Table 2). The top signal *MTHFR*-rs1801133 was not associated with obesity in both first and second phases or after meta-analysis (Tables 2 and 3).

We also assessed association of variants with quantitative traits related to type 2 diabetes and obesity. The first phase control subjects showed only nominal association with the quantitative traits as shown in Supplementary Table  5. Most significant association was observed at *AMD1*-rs1007274 with LDL-C levels (*β* = −6.35 (95% CI (−9.61)–(−3.1)), *P* = 1.41 × 10^−4^). *MTHFR* variant rs1801133 was nominally associated with 2h PPG (*β* = −5.06 (95% CI (−9.72)–(−0.40)), *P* = 0.03), HDL-C (*β* = 2.14 (95% CI 0.68–3.59), *P* = 0.004) and TC (*β* = 5.61 (95% CI 1.28–9.94), *P* = 0.01) in the first phase (Table 3). In the second phase, while none of the above associations were replicated, new associations were observed with FPI (*β* = −0.77 (95% CI (−1.42)–(−0.11)), *P* = 0.02), HOMA-IR (*β* = −0.20 (95% CI (−0.36)–(−0.04)), *P* = 0.01), HOMA-B (*β* = −652.8 (95% CI (−1165)–(−140.1)), *P* = 0.01) and HOMA-S (*β* = 0.62 (95% CI 0.13–1.11), *P* = 0.01). Meta-analysis revealed association of *MTHFR*-rs1801133 with LDL-C (*P*_*r*_/*P*_*f*_ = 0.02/0.02, *Q* = 0.81, *I* ^2^ = 0) and TC (*P*_*r*_/*P*_*f*_ = 0.01/0.03, *Q* = 0.26, *I* ^2^ = 22.03). The association analysis results of *MTHFR*-rs1801133 with all traits (dichotomous and continuous) in the initial and replication phases and after meta-analysis are presented in Table 3. However, none of the associations observed in the present study were significant after correcting for multiple testing.

## 4. Discussion

Homocysteine plays an important role in cell metabolism as it is involved as an essential intermediate in the transfer of activated methyl groups in the activated methyl cycle. This cycle is responsible for global and gene promoter-specific DNA methylation, an important factor in regulating gene expression [38–40]. The biological relevance of homocysteine metabolism and its association with metabolic disorders make it an important candidate pathway for type 2 diabetes. To the best of our knowledge, the present study is first to comprehensively evaluate association of variants of homocysteine metabolism pathway genes as well as genes associated with homocysteine levels with type 2 diabetes, obesity, and related traits.

The C677T (rs1801133) variant of *MTHFR* is an established variant for plasma homocysteine levels [20, 41] and has also been reported to be associated with type 2 diabetes, its complications, and related traits like LDL-C levels [42, 43]. Association of *MTHFR*-rs1801133 with type 2 diabetes in different population has been inconsistent [13, 17, 44]. The association studies of *MTHFR*-rs1801133 with type 2 diabetes and LDL-C levels have also been limited to studies with small sample sizes. In the present study, *MTHFR*-rs1801133 showed association with type 2 diabetes and 2h PPG, HDL-C, and TC in initial phase but could not be replicated in the second phase. Interestingly, *MTHFR*-rs1801133 showed nominal association with LDL-C levels in meta-analysis as also suggested in a previous study though this association was not significant after correcting for multiple testing. The effect of *MTHFR*-rs1801133 variant on type 2 diabetes is most likely to be modulated by influencing levels of homocysteine. However, in the absence of levels of homocysteine in the present study population, this aspect could not be probed.

We also observed association of other investigated variants with quantitative traits. Earlier reports have suggested variants from *MTR* to be associated with obesity [27], similar to this, we also observed variants of *MTR* (rs16834521) to be associated with BMI. Variants from this gene were also associated with TC (rs1805087 and rs10737812) and HbA1c (rs1805087) levels. Interestingly, for the first time, we found association of variant rs1007274 of *AMD1* with plasma levels of LDL-C, CRP and TC. Though these associations could not reach the threshold after multiple testing corrections, they provide important leads for follow-up studies in larger study populations. 

The present study is the largest among the studies investigating association of *MTHFR*-rs1801133 and was sufficiently powered to detect the previously reported effect sizes. However, we were unable to confirm the associations in Indian population. Differences in genetic architecture and LD pattern due to ethnic differences [17, 21, 44] could be one of the possible reasons for the observed inconsistency in the association of *MTHFR*-rs1801133 with type 2 diabetes. Previous studies by our group also observed inconsistency in association of variants of *TNF-LTA* [45] and *TNFRSF1B* [46] in Indians as compared to other ethnic groups indicating difference in genetic structure.

Population stratification may also give rise to spurious associations in a case-control study. However, cases and controls in our study populations were recruited from geographical locations that form a homogenous cluster as suggested by Indian genome variation consortium [47]. The multidimensional scaling (MDS) plot drawn from the information of 608 independent variants genotyped in the same set of samples indicates genetic homogeneity of the samples (Supplementary Figure  1). Study subjects of present study are also a part of an ongoing genome-wide association study of type 2 diabetes (unpublished data) and have been found to belong to single cluster after performing MDS using genome-wide SNP data. Hence, the subjects of two study populations were genetically similar, but phenotypic difference in terms of biochemical traits was observed between the two study groups which could have influenced association results. It is also known that dietary habits can influence levels of homocysteine and in Indians; individuals with vegetarian diet have been shown to have higher levels of homocysteine [48]. We observe difference in the number of individuals on vegetarian and nonvegetarian diets among both cases and controls of phase1 and phase2 (Supplementary Table  6). We believe this difference in status of vegetarian/nonvegetarian diet would have led to the difference in the association results of the two phases, but additional information on the frequency and kind of non-vegetarian diet along with other dietary information would have best answered this question.

## 5. Conclusions

In conclusion, variants from genes involved in homocysteine metabolism showed suggestive evidence for association with type 2 diabetes, obesity, and other related traits. However, these associations were not confirmed upon replication. Thus, studies involving a larger study population and different ethnic groups are required before completely ruling out the role of these important candidate genes in type 2 diabetes, obesity, and related traits.

## Figures and Tables

**Table 1:** Anthropometric and clinical characteristics of the study populations.

Characteristics	Phase 1		Phase 2	
	Type 2 diabetes patients	Control Subjects	Type 2 diabetes Patients	Control Subjects
*N* (Men/Women)	1019 (592/427)	1006 (606/400)	1047 (619/428)	1038 (516/522)
Age (years)	53 (45–62)	50 (44–60)	55 (49–62)	54 (45–64)
BMI (Kg/m^2^)				
Men	23.80 (22.00–26.00)	23.10 (20.10–5.70)	24.82 (22.68–27.76)	24.68 (22.35–27.39)
Women	26.70 (24.20–29.20)	25.00 (21.10–8.50)	27.39 (24.56–30.63)	26.37 (23.13–29.33)
WHR				
Men	1.00 (0.97–1.03)	0.97 (0.92–1.0)	0.98 (0.95–1.03)	0.97 (0.93–1.01)
Women	1.00 (0.97–1.03)	0.86 (0.82–0.92)	0.93 (0.87–0.97)	0.86 (0.82–0.90)
Systolic BP (mmHg)	130 (130–140)	120 (112–132)	130 (122–140)	130 (120–140)
Diastolic BP (mmHg)	80 (78–90)	80 (70–88)	82 (80–90)	80 (78–90)
HbA1c (%)	7.80 (6.50–9.40)	5.20 (4.90–5.60)	8.20 (6.90–9.60)	5.65 (5.33–5.89)
FPG (mmol/L)	7.90 (6.40–10.30)	4.90 (4.50–5.30)	7.79 (6.21–10.27)	4.87 (4.43–5.27)
2h PPG (mmol/L)		5.57 (4.83–6.30)		5.63 (4.88–6.22)
FPI (pmol/L)	82.80 (42.0–166.80)	32.40 (17.40–57.60)	74.40 (26.00–96.00)	43.80 (28.20–63.60)
HOMA-IR	5.20 (2.30–9.60)	1.20 (0.60–2.00)	4.30 (2.30–9.30)	1.60 (1.00–2.40)
HOMA-B	60.25 (25.3–154.51)	73.40 (40.70–138.60)	61.66 (25.61–131.86)	105.27 (65.13–167.32)
HOMA-S	0.20 (0.10–0.46)	0.86 (0.49–1.69)	0.23 (0.11–0.44)	0.64 (0.42–1.03)
C-peptide (nmol/L)	0.89 (0.56–1.36)	0.53 (0.36–0.73)	1.05 (0.69–1.60)	0.66 (0.50–0.86)
CRP (mg/L)	2.20 (0.90–4.70)	1.30 (0.60–3.00)	1.86 (0.90–3.44)	1.61 (0.90–3.04)
TC (mmol/L)	4.20 (3.50–5.00)	4.40 (3.7–5.10)	4.64 (3.86–5.42)	4.91 (4.22–5.52)
LDL-C (mmol/L)	2.57 (1.99–3.36)	2.79 (2.33–3.41)	2.73 (2.10–3.42)	3.01 (2.49–3.51)
HDL-C (mmol/L)	1.03 (0.88–1.22)	1.06 (0.88–1.28)	1.11 (0.94–1.34)	1.24 (1.06–1.46)
TG (mmol/L)	1.60 (1.10–2.20)	1.30 (1.00–1.80)	1.43 (0.98–2.13)	1.22 (0.86–1.64)

Values provided are median (interquartile range).

*N*: number of individuals, BMI: body mass index, WHR: waist to hip ratio, BP: blood pressure, HbA1c: glycosylated haemoglobin 1c, FPG: fasting plasma glucose, 2h PPG: 2h postload plasma glucose, FPI: fasting plasma insulin, HOMA-IR: homeostasis model assessments of insulin resistance, HOMA-B: homeostasis model assessments for *β*-cell function, HOMA-S: homeostasis model assessments for insulin sensitivity, CRP: C-reactive protein, TC: total cholesterol, LDL-C: low-density lipoprotein cholesterol, HDL-C: high-density lipoprotein-cholesterol, TG: triglyceride.

**Table 2:** Variants showing association (uncorrected *P*-value) with type 2 diabetes and obesity.

Trait	Gene	SNP	Allele (major/minor)	MAF (affected/unaffected)	OR (95% CI)	*P*
Type 2 diabetes (total study population: 1019 cases versus 1006 controls)^†^	*MTHFR*	rs1801133	C/T	0.18/0.21	0.78 (0.67–0.92)	0.003

Type 2 diabetes (Normal weight subjects: 290 cases versus 436 controls)^†^	*MTHFR*	rs1801133	C/T	0.18/0.21	0.62 (0.46–0.85)	0.002
	*MTHFR*	rs9651118	C/T	0.26/0.25	1.31 (1.01–1.68)	0.04
	*CHDH*	rs4563403	G/A	0.10/0.11	0.66 (0.45–0.95)	0.02
	*CBS*	rs706208	T/C	0.41/0.39	1.30 (1.03–1.63)	0.03
	*MTHFD1L*	rs1555179	C/T	0.26/0.23	1.36 (1.04–1.78)	0.03

Type 2 diabetes (Obese/over-weight subjects: 691cases versus 562 controls)^†^	*MTHFD1*	rs1076991	A/G	0.40/0.44	0.81 (0.69–0.95)	0.01
	*TCN2*	rs1801198	G/C	0.42/0.44	0.85 (0.72–0.99)	0.04

Obesity (562 Obese/over-weight versus 436 normal weights)^‡^	*CHDH*	rs4563403	G/A	0.09/0.13	0.69 (0.52–0.92)	0.01
	*TCN2*	rs1801198	G/C	0.46/0.41	1.24 (1.04–1.48)	0.02
	*MTR*	rs16834521	T/C	0.31/0.35	0.82 (0.68–0.99)	0.04

^†^Analyses adjusted for sex, age, and BMI. ^‡^Analysis performed only in control subjects and adjusted for sex and age, MAF: minor allele frequency, OR: odds ratio, CI: confidence interval.

**Table 3:** Association of rs1801133 with all traits in initial phase, replication phase, and meta-analysis.

Trait	Initial phase		Replication phase		Meta-analysis					
	OR*/*β**(95% CI)	*P*	OR/*β* (95% CI)	*P*	*P*_*f*_	*P*_*r*_	OR_*f*_/*β*_*f*_	OR_*r*_/*β*_*r*_	*Q*	*I*
Type2 diabetes (all)^ †^	0.78 (0.67–0.92)	0.003	1.01 (0.87–1.19)	0.86	0.05	0.38	0.90	0.89	0.024	80.44
Type2 diabetes (NW)^ †^	0.62 (0.46–0.85)	0.002	1.11(0.80–1.54)	0.53	0.07	0.52	0.81	0.83	0.012	84.30
Type2 diabetes (OW/OB)^ †^	0.86 (0.71–1.04)	0.13	0.98 (0.82–1.17)	0.85	0.25	0.25	0.93	0.93	0.32	0.25
Obesity (NW versus OW/OB)^ ‡^	1.07 (0.86–1.33)	0.56	1.11 (0.86–1.43)	0.42	0.33	0.33	1.09	1.09	0.82	0
BMI^‡^	0.32 ((−0.19)–(−0.84))	0.22	0.25 (−0.26–0.76)	0.34	0.12	0.12	0.29	0.29	0.84	0
Weight^‡^	1.41 (0.02–2.80)	0.05	0.43 (−0.95–1.81)	0.54	0.07	0.07	0.92	0.92	0.33	0
WC^‡^	0.47 (−0.76–1.70)	0.45	0.28 (−1.12–1.68)	0.70	0.41	0.41	0.39	0.39	0.84	0
WHR^‡^	−0.003 (−0.01–0.005)	0.46	−0.005 (−0.01–0.003)	0.24	0.18	0.18	−0.004	−0.004	0.73	0
FPG^†^	0.55 (−0.66–1.77)	0.37	-0.83 (−2.14–0.47)	0.21	0.84	0.86	−0.09	−0.12	0.13	56.98
2h PPG^†^	−5.06 ((−9.72)–(−0.40))	0.03	−1.13 (−4.84–2.58)	0.55	0.07	0.15	−2.65	−2.83	0.20	40.15
HbA1c^†^	0.02 (−0.03–0.07)	0.41	−0.03 (−0.07–0.01)	0.15	0.58	0.79	−0.009	−0.007	0.12	59.73
FPI^†^	0.43 (−0.44–1.30)	0.33	−0.77 ((−1.42)–(−0.11))	0.02	0.21	0.73	−0.33	−0.20	0.03	78.49
HOMA-IR^†^	0.11 (−0.09–0.31)	0.30	−0.20 ((−0.36)–(−0.04))	0.01	0.21	0.73	−0.08	−0.05	0.02	81.77
HOMA-B^†^	70.25 (−34.99–175.50)	0.19	−652.8 ((−1165)–(−140.1))	0.01	0.44	0.49	41.02	−245.94	0.0068	86.36
HOMA-S^†^	−0.09 (−0.26–0.07)	0.27	0.62 (0.13–1.11)	0.01	0.82	0.53	−0.02	0.23	0.0067	86.41
C-peptide^†^	0.01 (−0.10–0.12)	0.86	−0.07 (−0.17–0.04)	0.22	0.44	0.44	−0.03	−0.03	0.32	0
CRP^†^	−0.16 (−0.38–0.05)	0.14	−0.12 (−0.34–0.1)	0.28	0.07	0.07	−0.14	−0.14	0.79	0
HDL-C^†^	2.14 (0.68–3.59)	0.004	−0.05 (−1.31–1.21)	0.94	0.07	0.36	0.89	1.01	0.03	79.83
LDL-C^†^	3.25 (−0.32–6.81)	0.07	2.61 (−1.03–6.26)	0.16	0.02	0.02	2.94	2.94	0.81	0
TC^†^	5.61 (1.28–9.94)	0.01	2.02 (−2.43–6.47)	0.37	0.01	0.03	3.86	3.85	0.26	22.03
TG^†^	7.08 (−0.88–15.05)	0.08	−3.73 (−10.26–2.81)	0.26	0.81	0.79	0.62	1.43	0.04	76.37
Systolic BP^†^	1.53 (−0.41–3.48)	0.12	−0.77 (−2.58–1.03)	0.40	0.66	0.76	0.29	0.35	0.09	65.50
Diastolic BP^†^	0.77 (−0.30–1.85)	0.16	−0.74 (−1.82–0.35)	0.18	0.95	0.98	0.02	0.02	0.05	73.47
Creatinine^†^	0.02 (−0.004–0.04)	0.11	−0.01 (−0.05–0.02)	0.50	0.33	0.68	0.01	0.01	0.16	50.00
Urea^†^	0.28 (−0.63–1.20)	0.54	−0.12 (−1.14–0.9)	0.82	0.76	0.76	0.10	0.10	0.57	0
Uric acid^†^	0.37 (−0.02–0.76)	0.06	0.05 (−0.1–0.19)	0.55	0.22	0.31	0.09	0.15	0.13	56.68

^†^Analyses adjusted for sex, age, and BMI.

^‡^Analysis performed only in control subjects and adjusted for sex and age.

OR: odds ratio, CI: confidence interval, *P*_*f*_: *P* value for fixed effect, *P*_*r*_: *P* value for random effect OR_f_: odds ratio for fixed effect, *β*_f_: beta value for fixed effect, OR_r_: odds ratio for random effect, *β*_*r*_: beta value for random effect, *Q*: *P* value for Cochrane's *Q* statistic, and *I*: *I* ^2^ heterogeneity index (0–100).
